# The reference genome and organelle genomes of wasabi (*Eutrema japoniacum*)

**DOI:** 10.3389/fgene.2022.1048264

**Published:** 2022-10-31

**Authors:** Hexia Liu, Qinghua Qiao, Xiaoxia Ye, Yipeng Guo, Baojian Ye, Qiuyuan Xu, Xingwen Zhou, Bo Li

**Affiliations:** ^1^ College of Biology and Pharmacy, Yulin Normal University, Yulin, China; ^2^ College of Architecture and Urban Planning, Fujian University of Technology, Fuzhou, China

**Keywords:** wasabi, genome assembly, organelle genomes, nanopore, illumina

## Abstract

Wasabi (*Eutrema japonicum*) is one of the most famous vegetable crops in the family Brassicaceae. However, a limited genomic resource is available, which hinders genomic breeding and understanding of the genetic basis of vital traits. Here, we generated the genome assembly of wasabi using the hybrid genome assembly strategy, which combined the Nanopore long reads and Illumina reads. The genome assembly contains 687M bp and 39,534 high-quality annotated gene models. Besides, we annotated 68.85% of the genomic sequences as repetitive elements, including 43.72% of retrotransposons and 18.99% of DNA transposons. Using the customized pipeline, we also generated the complete organelle genomes of wasabi. This reference genome could provide essential genomic resources for evolution, breeding, and exploring the unique biological traits of wasabi.

## Introduction

Wasabi (*Eutrema japonicum*) is a perennial herb that plays a vital role in Japanese cuisine and culture (Yamane et al., 2016; Ramirez et al., 2020). A paste made from ground rhizomes is used as a pungent condiment, similar to hot mustard or horseradish (Ramirez et al., 2020; Yang et al., 2020). Wasabi is a plant of the family Brassicaceae (Yamane et al., 2016). Brassicaceae is a medium-sized and economically important family of flowering plants commonly known as the mustards, the crucifers, or the cabbage family (Guo et al., 2017; Nikolov et al., 2019). The family contains cruciferous vegetables, including species such as *Brassica oleracea* (cabbage), *Brassica rapa* (turnip), *Brassica napus* (rapeseed), *Raphanus sativus* (radish), *Armoracia rusticana* (horseradish), but also a cut-flower Matthiola (stock) and the model organism *Arabidopsis thaliana* (Guo et al., 2017; Nikolov et al., 2019). Despite its high economic value, only a few genomes from the family have been deciphered, e.g., *Brassica oleracea*, *Brassica rapa,* and *Arabidopsis thaliana* (Wang et al., 2011; Liu et al., 2014).

*Eutrema* is a genus in the Brassicaceae that contains more than 30 species, most of which are distributed in eastern Asia, e.g., the model plant *E. salsugineum*, which is used for studies of abiotic stress. Most of these species occur in the Qinghai-Tibet Plateau (QTP). Several studies have been conducted to understand the genomes of *Eutrema*. Yang et al. (2013) presented the reference genome sequence (241 Mb) of *E. salsugineum* at 8 × coverage sequenced using the traditional Sanger sequencing-based approach. Guo et al. (2018) provide *de novo* whole-genome assemblies for a pair of recently diverged perennials with contrasting altitude preferences, the high-altitude *E. heterophyllum* from the eastern Qinghai-Tibet Plateau and its lowland congener *E. yunnanense*.

As for the high commercial crop, the chloroplast genome sequence of wasabi and its relatives were decoded to understand the evolution and phylogeny (Haga et al., 2019). Besides, the high repeat sequence content in its relative species also suggests the potential high repeat in the wasabi genome (Guo et al., 2018). This study performed hybrid genome *de no* assembly towards wasabi, combining Nanopore long reads with illumine short reads. After detailed genome annotation, we obtained a high-quality dataset of the wasabi genome, providing us with a powerful tool to conduct related genomic studies and breeding programs.

## Materials and methods

### Plant materials

The plant material was obtained from the experimental plantation in Liangshan (27.88°N, 102.26°E), Sichuan, China. We selected a well-planted *Eutrema japoniacum* sample for all experiments. Three different tissues (leaf, root, and stem) were collected for RNA sequencing. Fresh and healthy samples were harvested and frozen in liquid nitrogen immediately after collection and stored at −80°C in the laboratory.

### Whole genome sequencing and RNA sequencing

Genomic DNA was extracted with a QIAGEN^®^ Genomic Kit from leaves. The purified DNA was then prepared using the SQK-LSK109 genome sequencing kit protocol [Oxford Nanopore Technologies (ONT), Oxford, United Kingdom]. Single-molecule real-time sequencing of long reads was performed on a PromethION platform (ONT, Oxford, United Kingdom). Additionally, the Illumina sequencing libraries were constructed following the manufacturer’s instructions. The libraries with an insert size of 300 bp were created using the TruSeq Sample Preparation kit and sequenced on the Illumina HiSeq X-ten platform (Illumina, San Diego, CA).

The samples of different tissues (leaf, root, and stem) were prepared for library construction. The quality and quantity of the RNA were evaluated using a NanoDropTM D-1000 spectrophotometer (NanoDrop Technologies, Wilmington, DE), a Qubit^®^ 3.0 Fluorometer (Thermo Fisher Scientific, United Stated), and an Agilent Bioanalyzer 2100 (Agilent Technologies, CA, United States). The constructed libraries were sequenced using the similar method above.

### Genome survey, genome assembly, and genome annotation

We counted the *K*-mers using Jellyfish software and calculated the characteristics of the genome using Genomescope 2.0 software (Vurture et al., 2017). We performed genome assembly using MaSuRCA software (Zimin et al., 2013), which adopted a hybrid strategy. Genome annotation was conducted using the Maker-P pipeline (Campbell et al., 2014). Using the RNA-seq reads from different libraries, we performed *de novo* assembly with SOAPdenovo-Trans (Xie et al., 2014). To identify other kinds of repeat sequences in the wasabi genome assembly, we first built a non-redundant repeat sequence library by searching for repetitive sequences using the EDTA pipeline (Ou et al., 2019). The tRNA genes were predicted using the tRNAscan-SE package (version 1.3.1) with default parameters (Chan et al., 2021). The rRNA genes (8S, 18S, and 28S) were predicted using RNAmmer algorithms with default parameters (Lagesen et al., 2007). The miRNA and snRNA were identified using INFERNAL (version 1.1.3) by searching against the publicly available Rfam database (release 13.0) (Griffiths-Jones et al., 2003).

### Assessment of assembly quality

We assessed the completeness of wasabi genome assembly using BUSCO software (version 4.0.5) with the parameters “-l embryophyta_odb10 -g genome” (Simão et al., 2015). We performed a sequence identity assessment by aligning the non-redundant Illumina short reads to the wasabi genome assembly using BWA with default parameters (Li and Durbin, 2009). Finally, RNA-seq data of different tissues were aligned to the wasabi genome using Hisat2 with default parameters (Kim et al., 2019).

### Gene functional annotation

To annotate the genes, we identified the homologous genes with the National Centre for Biotechnology Information (NCBI) Non-Redundant (NR) Protein Database, the Universal Protein Resource Knowledgebase (UniProtKB), Swiss-Prot Protein Database using BLASTP with an E-value threshold of 1e-5 (Altschul et al., 1990). Functional domains were identified using InterProScan (version 5.2–45.0) against several publicly available databases (Blum et al., 2021). Metabolic pathway annotations were performed by sequence comparisons with the Kyoto Encyclopaedia of Genes and Genomes (KEGG) database (release 92.0) using BLASTP with an E-value of 1e-5 (Kanehisa et al., 2021).

### *De novo* assembly of organelle genome

Firstly, we used the conserved gene sequences from organelles as baits files to extract Nanopore organelle reads including mitochondria and chloroplast. Secondly, the long reads were assembled using Flye software (Kolmogorov et al., 2019) and polished by Racon software (Vaser et al., 2017). The assemblies were used as the new baits file to re-extract nanopore organelle reads and Illumina reads. Finally, the Unicycler software (Wick et al., 2017) was used to conduct the organelle genomes. Annotation was performed with GE-Seq using default parameters to predict protein-coding genes, tRNA genes, and ribosomal RNA (rRNA) genes (Tillich et al., 2017). Manual annotation was performed for genes with low sequence identity to determine the positions of start and stop codons depending on the translated amino acid sequence using the chloroplast/bacterial genetic code. OrganellarGenomeDRAW (OGDRAW) was optimized to create detailed, high-quality maps of organellar genomes (Greiner et al., 2019).

## Results and discussion

### Genome survey and genome assembly

To obtain the wasabi genome assembly, we generated a set of 122-fold-coverage Illumina paired-end short reads (75.62 Gb) and 30-fold-coverage Nanopore long reads (26.83 Gb) (Supplementary Table S1). We firstly conducted the genome survey using the *K*-mer analysis. The wasabi genome size and the genome heterozygosity rate were estimated to be 640 Mb and 0.68%, respectively (Supplementary Figure S1). We assembled the reads into 687 Mb of contig sequences using the hybrid genome assembly strategy (Supplementary Table S2). We noticed that the size of genome assembly is slight larger that the estimated genome size, which may be caused by the assembly duplications or the underestimation of the genome survey. The genome assembly contains 7,875 contigs with an N50 length of 356,067 bp, and the largest contigs were 5,563,810 bp (Supplementary Table S2). We identified 90% of BUSCO gene models in the wasabi genome assembly (Supplementary Table S3), suggesting the completeness of the genome assembly. We furthermore remapped the sequencing reads to the genome to investigate the completeness. Nearly 96% of the DNA Illumina reads, and 99% of the Nanopore reads could be mapped to the genome. Moreover, an average of 80% of RNA-seq reads were aligned to the genome (Supplementary Table S4). All of the statistics reflected the relative completeness of the wasabi genome.

### Genome annotation and gene family analysis

**FIGURE 1:**
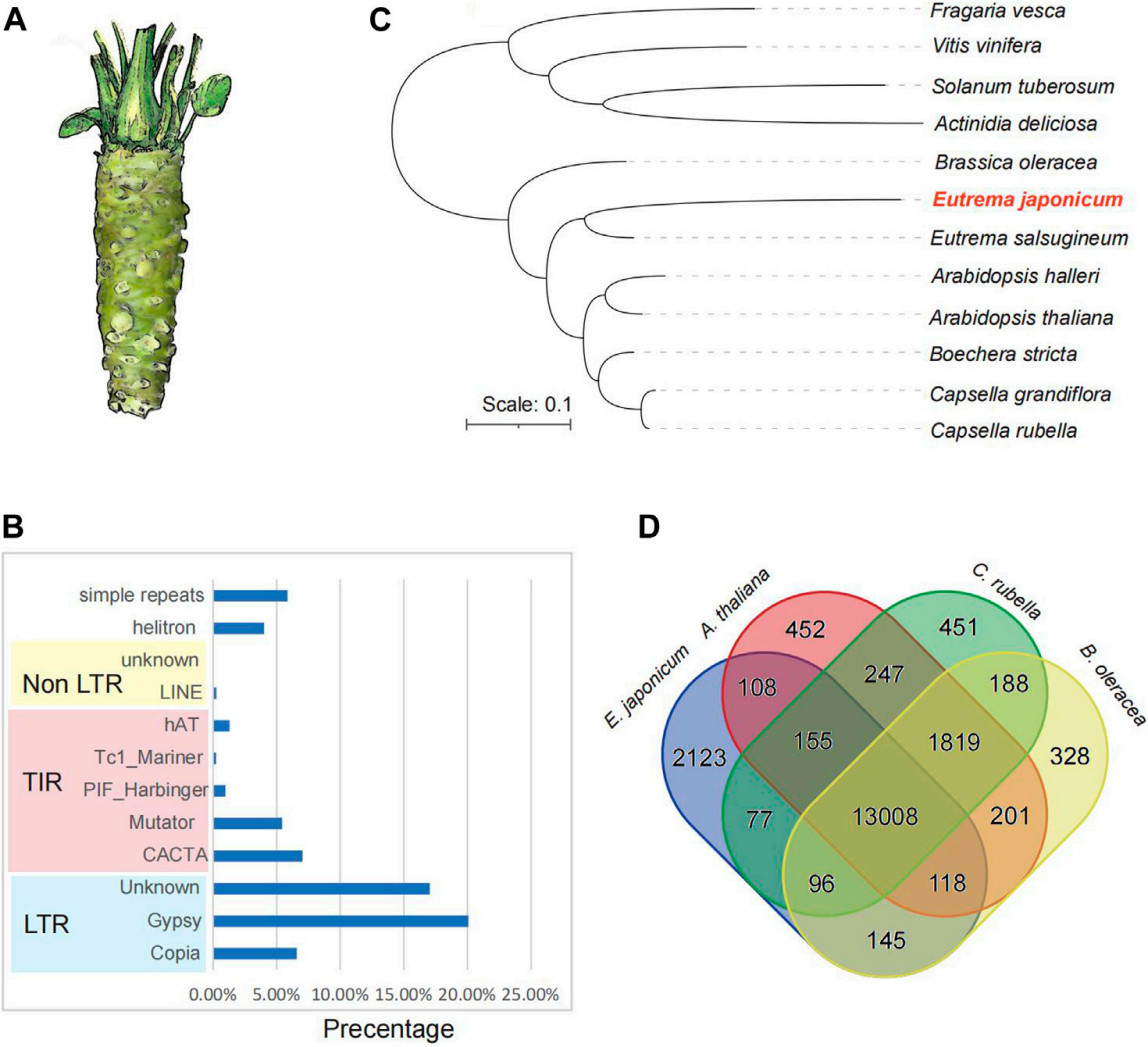
The genome assembly of wasabi (*Eutrema japoniacum*). **(A)** The morphology of the wasabi. **(B)** The statistics of repetitive sequence across the genome. **(C)** The phylogenetic tree of 12 representative species was constructed using the single-copy genes. **(D)** Veen diagram of orthologous groups from the four representative species: *Eutrema japoniacum*, *Arabidopsis thaliana*, *Brassica oleracea,* and *Capsella rubella*.

We first generate the high-quality library using the EDTA pipeline to produce a high-quality annotation of the repetitive sequences. We identified 9,042 intact repetitive sequence elements in the genome, including 6,241 LTR-RTs and 3,161 DNA transposons (Supplementary Table S5). Specifically, there were 1,302 *Copia* type-, 2583 Gypsy type- and unknown type-LTR-RTs (Supplementary Table S5). In the wasabi genome, repetitive sequences occupied 473.7 Mb (68.85% of the 687.9 Mbp). The most abundant repeats in this genome are retrotransposons (43.72% of genome assembly) (Figure 1B). The non-LTR retrotransposons (LINE and SINE) accounted for 0.27%. 18.99% of the genome sequences belong to the DNA transposons (14.99% for TIR and 4.00% for Helitrons) (Supplementary Table S6).

Using the RNA-seq data, we obtained a total of 120,945 transcripts. After the genome annotation, A total of 39,534 genes were predicted, 99.9% (39,495) of which could be functionally annotated referring to the currently available databases, and 84.2% (33,279) were expressed in at least one tissue (Supplementary Table S7). These results would provide valuable genetic resources for future functional genomics and molecular breeding research.

We analyzed the gene families using the genomes of wasabi and other 11 other representative plant species, including *Fragaria vesca*, *Vitis vinifera*, *Solanum tuberosum*, *Actinidia deliciosa*, *Brassica oleracea*, *Eutrema salsugineum*, *Arabidopsis halleri*, *Arabidopsis thaliana*, *Boechera stricta*, *Capsella grandiflora* and *Capsella rubella*. Three hundred seventy-seven thousand three hundred one genes were clustered into 27,255 orthologous groups (Supplementary Table S8). Seven thousand one hundred ninety-six groups were present in all 12 species, with 381 species-specific orthologous groups (Supplementary Tables S8, S9). We identified a total of 1,030 wasabi specific orthologous groups containing genes with a range of biological processes and 70% of them were annotated (Supplementary Table S10). The phylogenetic tree constructed using the single-copy genes indicated the phylogenetic position of the wasabi (Figure 1C).

### Organelle genomes

We conducted the organelle genomes using the customized pipeline, which integrated the Nanopore sequencing data and the Illumina data. The Nanopore long reads from the organelle genomes were fist conducted assembly to obtain the raw assembly. The Nanopore reads and Illumina reads with high-accuracy were integrated to obtain the final assembly. The customized pipeline could take advantage of the continuity of long reads and the high-accuracy of short reads. As a result, the length of the chloroplast genome was 153,847 bp and was circular, suggesting the completeness of the chloroplast assembly (Supplementary Table S11 and Supplementary Figure S2). The chloroplast genome exhibited a typical quadripartite structure, consisting of a pair of inverted repeat regions (IRs) (26,016 bp) separated by a large single copy region (LSC) (69,846 bp) and a small single copy region (SSC) (17,810 bp). One hundred seventy-seven genes were successfully annotated, containing 98 protein-coding genes, 49 tRNA genes, and 30 rRNA genes. As for the mitochondrion genome assembly, we obtained two independent contigs totaling 195,762 bp. The length of the longer contig was 127,834 bp, while the short one was 67,928 bp. After the detailed genome annotation, there were 166 genes, including 88 protein-coding genes, 16 rRNA genes, and 49 tRNA genes (Supplementary Table S11 and Supplementary Figure S3). We conducted the phylogenetic of both the organelle genomes with relative species indicated the phylogenetic position of the wasabi.

## Data Availability

The genome assembly data and all the raw sequencing data (including ONT sequencing, Illumina, and RNA-seq) are accessible through the China National Center for Bioinformation (https://ngdc.cncb.ac.cn/gsa/) under accession PRJCA012228. These supporting data (genome assemblies, gene annotations, and gff files for gene models) are also available at Figshare (https://doi.org/10.6084/m9.figshare.21286074.v2).
