# Supplementary material for: The reference genome and organelle genomes of wasabi (Eutrema japoniacum)
Source: Front Genet. 2022 Oct 31;13:1048264. doi: 10.3389/fgene.2022.1048264 (PMC9661192; doi:10.3389/fgene.2022.1048264)
Supplement: Supplementary file 1 [file DataSheet1.doc]

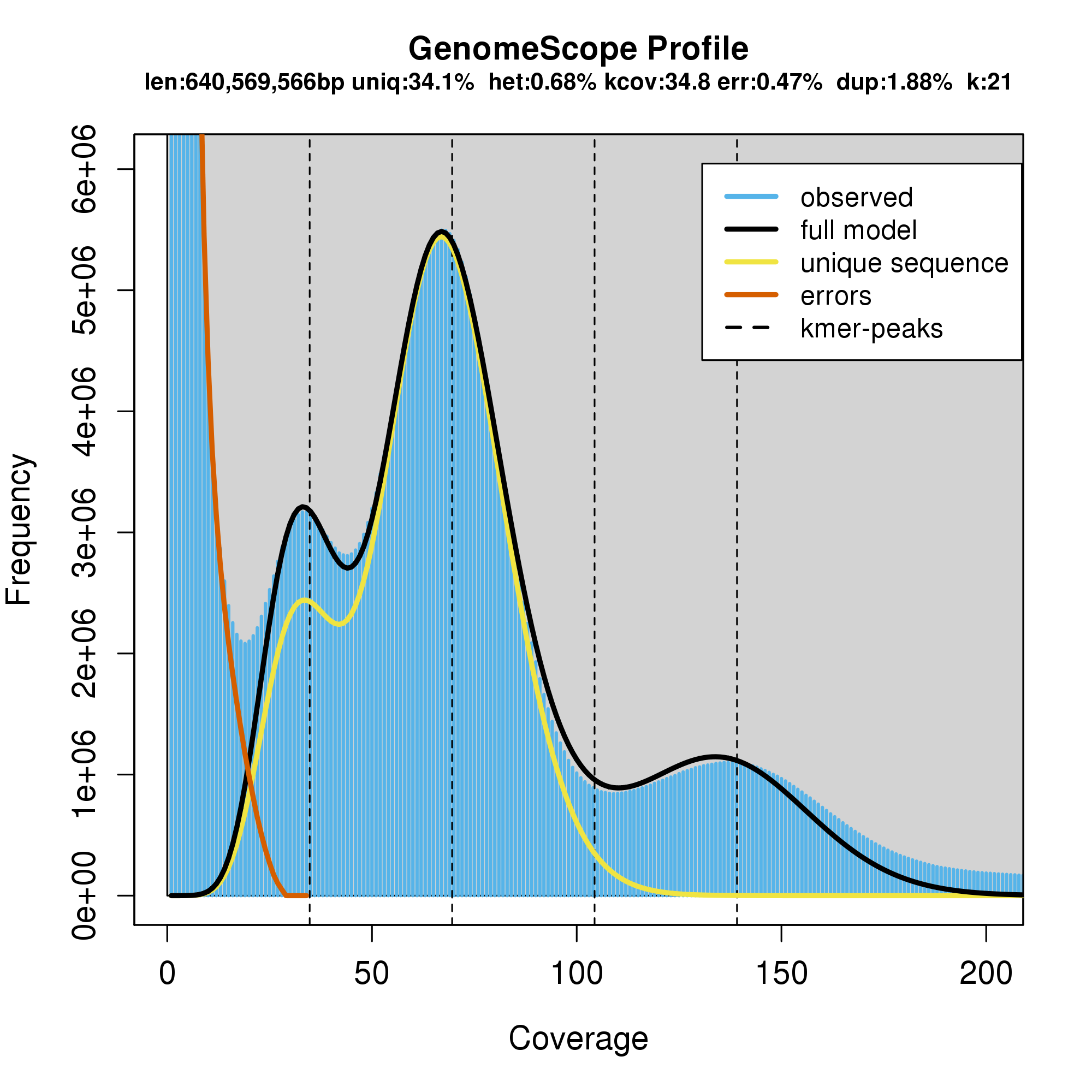


**Figure S1. The genome estimation of wasabi using GenomeScope 2.0**


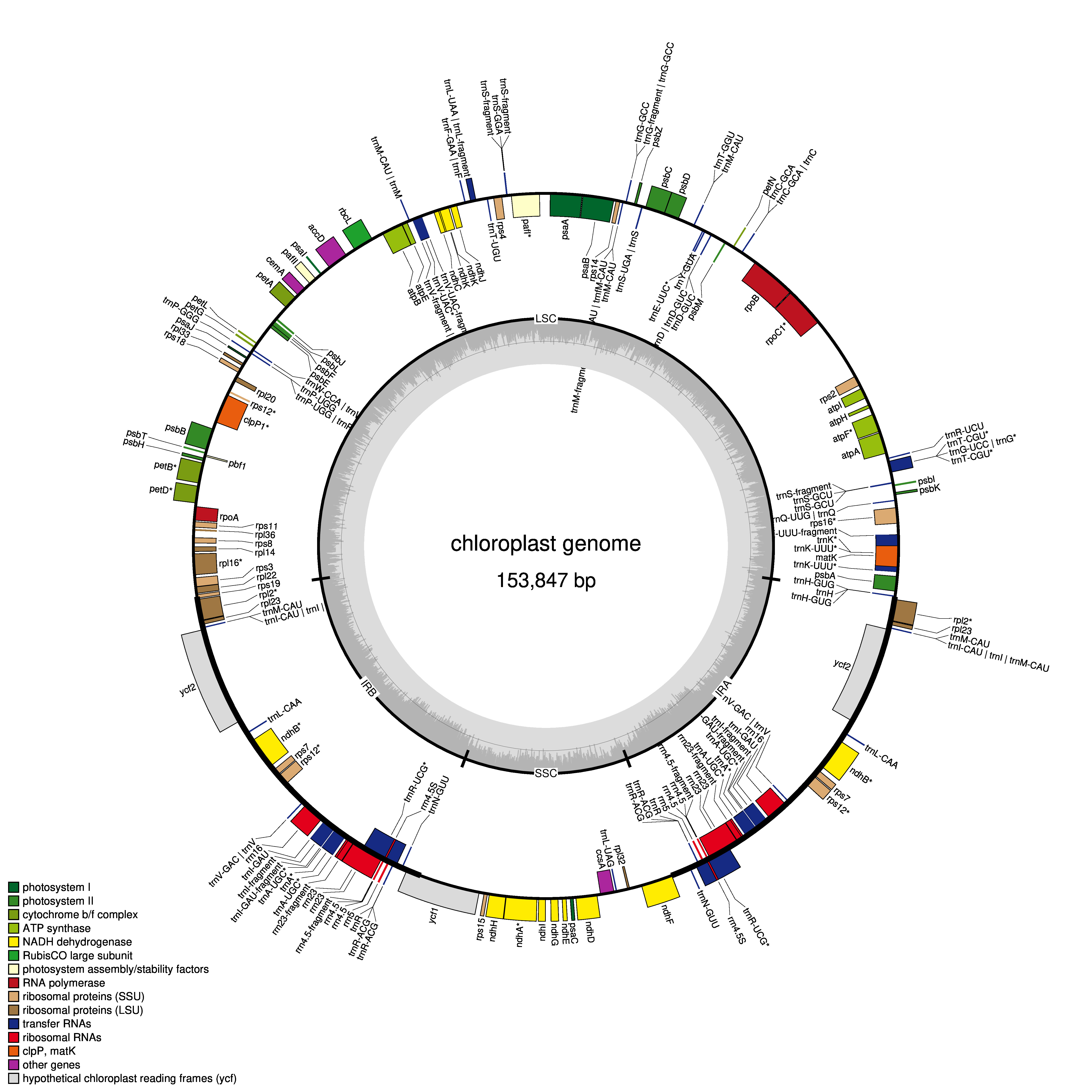


**Figure S2. Gene map of the chloroplast genome of wasabi**


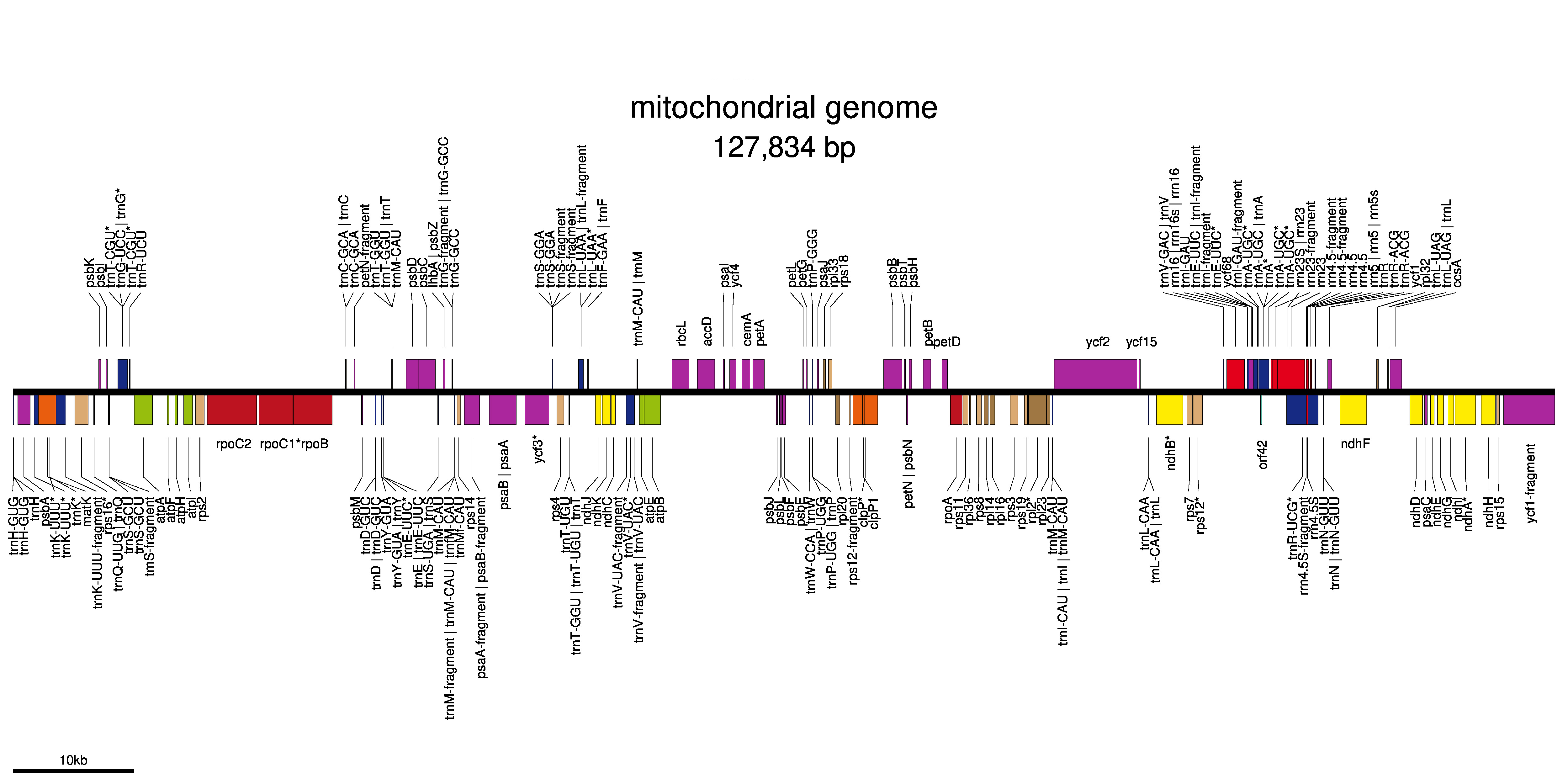


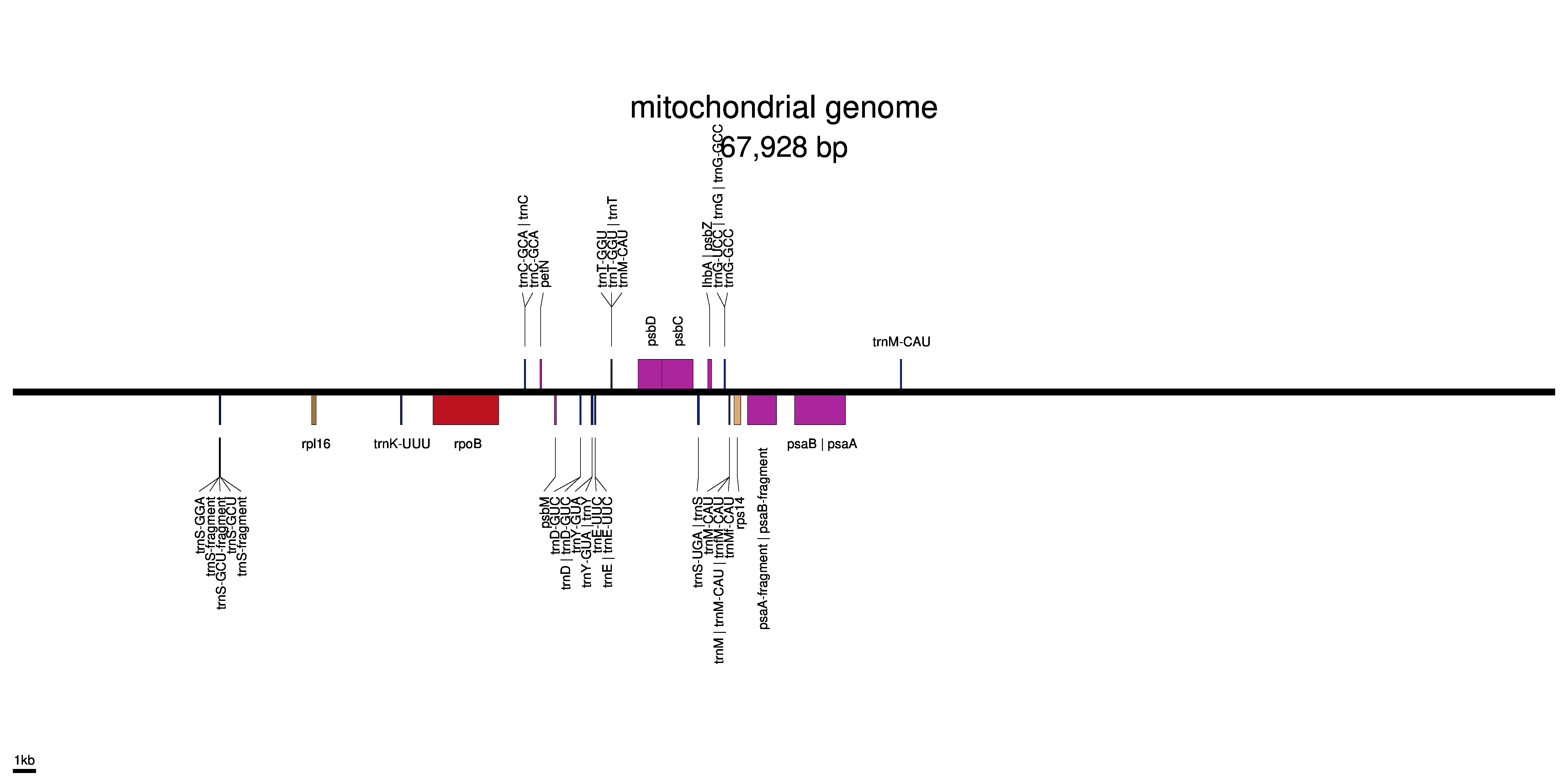


**Figure S3. Gene map of the mitochondrion genome of wasabi**

**
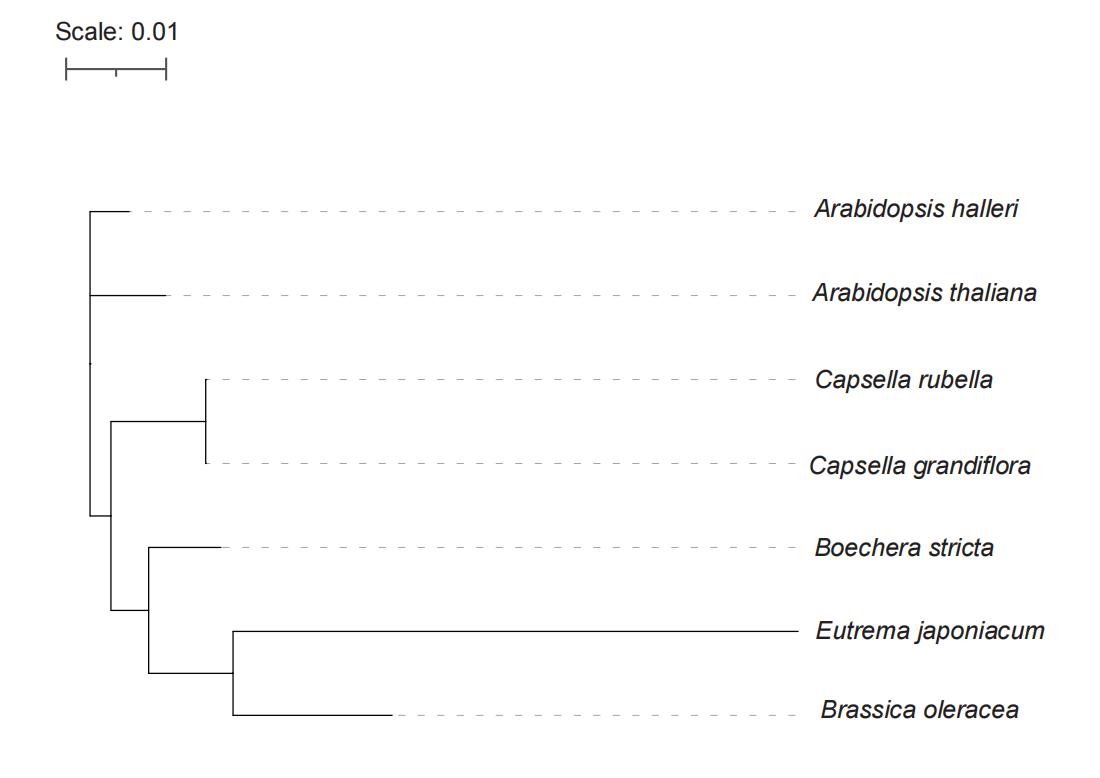
**

**Figure S4. Phylogenetic tree constructed by the chloroplast genomes**

**
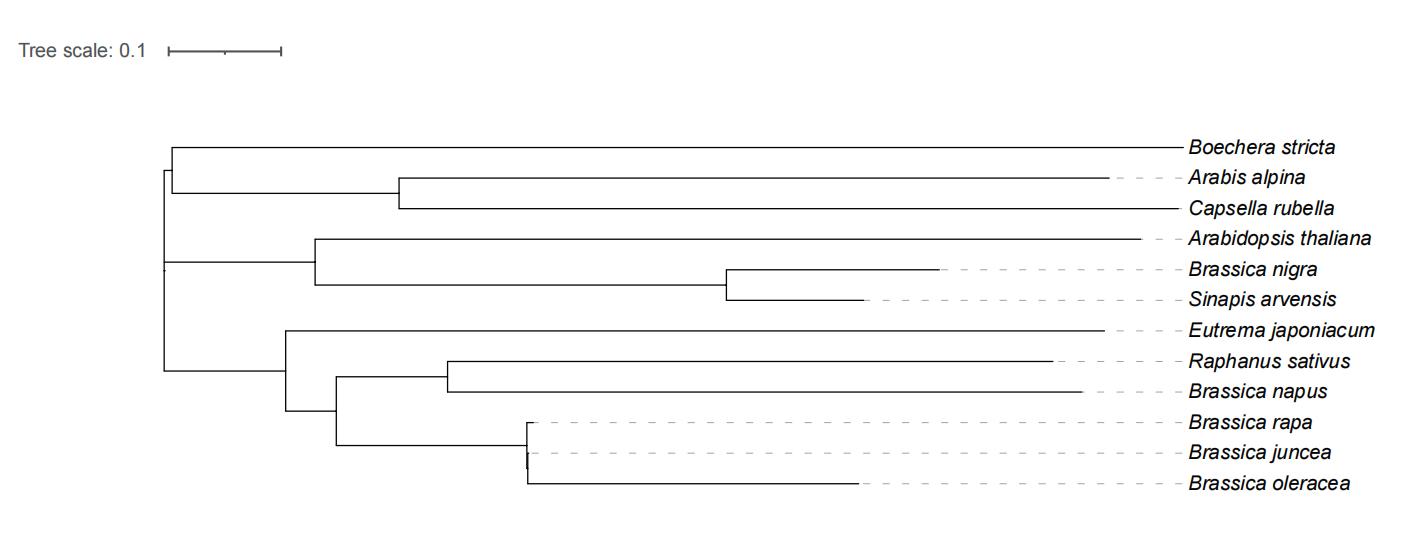
**

**Figure S5. Phylogenetic tree constructed by the mitochondrion genomes**
